# In and out Patients' Payments

**Published:** 1905-04-22

**Authors:** 


### The Hospital.

A Journal of

The Medical Sciences and Hospital Administration.

Vol. XXXVIII.—No. 969. SATURDAY, APRIL 22, 1905.

### In and Out Patients' Payments.

There can be no doubt that hospital opinion in favour of some system of payment by patients in hospitals in this country is increasing. This was made clear in the course of the debate on payment by out-patients and in-patients at the meeting of the Hospital Officers' Association on the 14th inst. Mr. Sydney Holland, who has borne the brunt of the criticism levelled at the plan whereby out-patients are enabled to contribute threepence towards the cost of medicine or bandages, made out a good case so far as the Poplar and the London Hospitals are concerned. Last year, at the London, 157,000 out-patients readily paid threepence, and out of 27,000 more, who claimed exemption on the ground of poverty, it was found on investigation, that 4,500 could pay and did pay. At the Poplar Hospital, where the patients belong almost entirely to the artisan class, only one in 90 of the patients declined or were unable to pay the threepence. These figures may be accepted as conclusive so far as the attitude of the patients is concerned. The financial criticism was in effect that seeing that the out-patients at the London cost about £20,000 a year, the £2,000 received from the out-patients' payments is an immaterial sum. As a matter of business no man could say that 10 per cent. is so small a saving as to be immaterial. From this point of view Mr. Sydney Holland had undoubtedly the best of the argument.

However, these threepenny payments by out-patients only touch the fringe of the question. That is probably why the general practitioners, and the consulting medical officers of hospitals in sympathy with them, are opposed to the system. Any system which enforces some payment by out-patients should be acceptable to the medical profession, because it must be better to teach the people to pay a little for medical relief, rather than to receive it for nothing. Mr. Ryan touched the kernel of the controversy when he asked for enlightenment, as to how the hospitals could be organised so as to confine free medical relief, to the large class of patients who cannot afford to pay for it elsewhere, and to them alone? A system which would secure this result would satisfy everybody. In our view the solution lies in each hospital so organising its work as to see each applicant once, and then to refer the patient to a provident or free dispensary working in co-operation with the hospital. On this plan it should be easily possible to limit the number of out-patients, to restrict free medical relief to deserving cases only, to ensure that the needs of the poorer classes, when ill, are adequately met by saving them the great loss of time entailed by the present system, and finally to provide that every poor sick person may readily obtain adequate medical attendance in his own home for nothing, if that be justifiable, or for a fee which he can easily pay. Arrangements under this system could be made, which would supply all the clinical material necessary for the purposes of medical instruction, which would fully protect the rights of the general practitioners who at present have good grounds for complaint against the hospitals, owing to the ever-increasing number of patients admitted to treatment, and so justice and efficiency might be secured for all the interests involved. We are further of opinion, that the claims of the poor, and the efficient working of such a system would be materially aided, by extending the district nursing associations to an adequate extent throughout the metropolitan area. By this means an accurate knowledge of the actual circumstances of each poor family would be made more readily and accurately obtainable, and the comfort and medical treatment of the sick members of their families could be immeasurably improved.

There remains the question of payment by in-patients. It was generally admitted, in the course of the debate, that the in-patient department is at present abused. It is rightly maintained, that those patients who are willing to pay, and can afford to pay something for their treatment in a hospital, should be afforded the privilege of doing so. Mr. Thies explained the continental system which lays it down that every patient in a hospital shall be paid for. The regulations provide that the poorest shall be paid for at a fixed rate by the municipal or county authorities, and that the accommodation for other paying patients shall be so arranged as to offer three scales of payment, so that the circumstances of all the patients may be adequately weighed and provided for. There can be little doubt that before many years are over every great hospital in this country must have paying wards as a part of its hospital provision. These paying wards should be provided in a separate wing, which would be under the control and management of the hospital authorities, but would be so situated as to allow the private medical attendant to visit his patient there without going into the hospital proper. All the patients' payments could then be pooled, and a percentage of them set aside for the remuneration of the practitioners in attendance. It would have the further advantage of enabling the nurses to be trained for private work, which it is not possible for the nurse-training schools to arrange to do adequately, under present circumstances. Good service has been done by imposing the small fee for medicine, because it has given an impetus to the demand for a reconsideration of the present system of free medical relief, which cannot continue unreformed for many years longer. By the co-operation of all the agencies, which at present give medical relief to the population of London, and by the institution of pay wings and a graduated scale of payments for all hospital patients, it should be possible, in the course of a few years, to provide, that whereas everybody who is entitled to it can readily obtain free medical relief, all other people will have to pay for it according to their means, under conditions, which will best minister to their comfort and rapid recovery.

